# Intersection of HIV and Reproductive Health

**DOI:** 10.1155/2013/418918

**Published:** 2013-11-24

**Authors:** Craig R. Cohen, Elizabeth Bukusi, Helen Rees, Kelly Blanchard

**Affiliations:** ^1^Department of Obstetrics, Gynecology and Reproductive Sciences, University of California, San Francisco, 50 Beale Street, San Francisco, CA 94105, USA; ^2^Centre for Microbiology Research, Kenya Medical Research Institute, Nairobi 00200, Kenya; ^3^Wits Reproductive Health and HIV Institute, University of Witwatersrand, Hillbrow, Johannesburg 2001, South Africa; ^4^Ibis Reproductive Health, Cambridge, MA 02138, USA

The HIV epidemic is integrally linked to reproductive health. Indeed HIV itself, which is predominantly a sexually transmitted infection, is a key reproductive health issue. In women, HIV can have adverse impact on pregnancy, childbirth, and breastfeeding. HIV status also affects conception and parenting choices. Both HIV and poor reproductive health share common drivers, including poverty, gender inequality, and social marginalization of vulnerable populations [1]. Responses to both health issues should therefore be closely linked and mutually reinforcing. The 2006 Political Declaration on HIV/AIDS that called for greater linkage between HIV/AIDS and reproductive health as an additional approach to curb the epidemic [2].

Furthermore, new approaches are needed to ensure that long-term, high-quality health services can meet both the HIV and broader reproductive health needs of women and men [3]. These innovative approaches include integrated services or a “one-stop shop,” where both services are provided in a single clinic with the same health provider during a single visit. As we approach the deadline for meeting the Millennium Development Goals in 2015 [4] and as the global health community debates the new Sustainable Development Goals for the post-2015 era [5], it is clear that greater integration of HIV and reproductive health research and service provision will be critical to achieve these goals.

The intersection between family planning and HIV services for women living with HIV is a central theme of this special issue. An estimated 13 million HIV-infected women live in sub-Saharan Africa [6]. Improved access to family planning among HIV-infected women will decrease maternal morbidity and mortality and improve neonatal outcomes [7]. Yet among HIV-infected women in this region, unintended pregnancy has been reported to range from 62% to 93%, including women on antiretroviral therapy [7, 8]. Despite current guidelines and best practice, contraceptive provision is not routinely offered as part of HIV services [9]. Even when it is offered, women and men frequently do not have information about or access to the full range of effective methods, and long-acting methods like intrauterine devices and contraceptive implants are often unavailable in high-HIV prevalence settings [10]. In addition, more attention is needed for drug interactions between antiretroviral drugs and hormonal contraceptives and for promotion of dual-method use. HIV-positive and -negative women need better information about and greater access to proven contraceptive methods.

For HIV negative women, there is a need to integrate HIV counselling and testing with contraceptive services, so that access to both services is increased. While pregnancy is known to increase the risk of HIV acquisition, antenatal services often fail to offer male or female condoms and safer sex counselling to pregnant women. In countries where there are high rates of teenage pregnancy and of HIV among adolescent girls, there are very few examples of effective integrated services for young women.

We hope that the papers in this special issue will bring additional attention to the intersection of HIV and reproductive health. A renewed focus on this intersection by populations, public and private health systems, country ministries of health and finance, and international donors, as well as attention by the larger global health research community, remains essential to continue to advance women's health. Such attention can also help promote gender equality, especially for those affected by and at risk of HIV. Readers will find a number of papers on providing comprehensive sexual and reproductive health services, including abortion care, to HIV-infected women from sub-Saharan Africa and Asia, prevention of gender-based and intimate partner violence, and the need to develop female-controlled multipurpose technologies designed to prevent pregnancy, HIV, and STI transmission.
